# Photodynamic Inactivation Effectively Eradicates *Candida auris* Biofilm despite Its Interference with the Upregulation of *CDR1* and *MDR1* Efflux Genes

**DOI:** 10.3390/jof8111137

**Published:** 2022-10-27

**Authors:** Matúš Štefánek, Lucia Černáková, Jaroslava Dekkerová, Helena Bujdáková

**Affiliations:** Department of Microbiology and Virology, Faculty of Natural Sciences, Comenius University in Bratislava, 842 15 Bratislava, Slovakia

**Keywords:** *Candida auris*, photodynamic inactivation, biofilm, efflux genes

## Abstract

*Candida auris*, in recent years, has emerged as a dangerous nosocomial pathogen. It represents a challenge for effective treatment because of its multiresistance. Photodynamic inactivation (PDI) is a promising way to solve problems with a wide range of resistant microorganisms. This study aimed to use PDI for the eradication of *C. auris* biofilms. Moreover, the regulation of the *CDR1*, *CDR2*, and *MDR1* resistance genes was studied. Experiments were performed on 24 h biofilms formed by three clinical isolates of *C. auris in vitro*. PDI was performed in the presence of the photosensitizer methylene blue (0.25 mM) and samples were irradiated with a red laser (λ = 660 nm, 190 mW/cm^2^) for 79, 120, and 300 s. To confirm the PDI effect, confocal laser scanning microscopy was performed after treatment. Effective PDI was achieved in all strains. The highest inhibition was observed after 300 s irradiation, with over 90% inhibition compared with the non-irradiated control sample. PDI was observed to upregulate the expression of the *CDR1* gene, but mainly the *MDR1* gene. Despite this observation, PDI significantly decreased the survival of *C. auris* biofilm cells and proved to have great potential for the eradication of problematic resistant yeasts.

## 1. Introduction

The emergence of resistant *Candida* species (spp.) poses a serious health risk, mainly for hospitalized patients. *C. auris* was already described to cause illness in patients more than a decade ago; nevertheless, its surge in incidence at the global level is alarming [1,2]. The majority of clinical isolates of *C. auris* manifest resistance to one or more main classes of antifungals—azoles, echinocandins, and polyenes [3,4,5]. Further complications are promoted by *C. auris* thermotolerance, osmotolerance, and resistance to disinfectants [6]. One of the major reasons that the medication of candidiasis failed, especially with azoles, is an increased activity of the efflux pumps. Two main classes of proteins are known to contribute to enhanced efflux in *Candida* spp.: ABC (ATP-binding cassette, Cdr1, Cdr2) and the MFS (major facilitator superfamily, Mdr1) [7,8,9].

It is generally known that the expression of efflux pumps is upregulated in fungal biofilms upon exposure to azoles, resulting in resistance and treatment failure [9]. Kean et al. (2018) showed that *C. auris* biofilm’s intermediate and mature stages differed from the initial phase of development as well as from the planktonic form by the upregulation of genes encoding efflux pumps including ABC and MFS transporters [10].

Nowadays, photodynamic inactivation (PDI) is a developing approach for fighting pathogenic microorganisms, including those manifesting multiresistance. PDI is based on the activation of a photosensitizer (PS) with light of an appropriate wavelength that induces cell death by apoptosis and/or necrosis. PSs should promote the formation of reactive oxygen species (ROS) followed by the oxidation of biomolecules [11,12,13,14]. Methylene blue (MB), one of the most commonly used PSs in antimicrobial PDI, has been playing important roles in microbiology and pharmacology and, lately, it has been considered a drug for PDI [15,16]. The main hypothesis of this manuscript is based on the questions of (i) whether and in what way PDI is related to the activity of efflux pumps and (ii) whether the effectiveness of PDI could work despite the activity of efflux transporters. These questions are legitimate, as a previous study conducted on *C. albicans* proved that the efflux of MB from the cytoplasm is associated exclusively with ABC transporters, while both the influx and efflux of MB can be regulated by an MFS system as well [17]. Despite this observation, other works focused on the regulation of efflux transporters have not been published and there is still a gap in this area of *C. albicans* research. Moreover, no studies have been published about the molecular response of *C. auris* biofilms to PDI treatment.

Therefore, the aim of this research was to provide a molecular analysis of the regulation of the genes coding for efflux; namely *CDR1*, *CDR2*, and *MDR1,* in the biofilm of resistant *C. auris* isolates with respect to the effectiveness of PDI. These data should contribute towards elucidating the interference of PDI with efflux in azole-resistant *Candida* spp.

## 2. Materials and Methods

### 2.1. Characterization of C. auris Isolates and Formation of 24 h Biofilm

This study worked on three clinical isolates of *C. auris*. *C. auris* H261 was kindly provided by prof. Birgit Willinger (Medical University, Vienna, Austria) and fluconazole (FLU)-resistant *C. auris* S and *C. auris* R were kindly provided by Prof. Elisa Borghi (Università degli Studi di Milano, Department of Health Sciences, San Paolo Medical School, Via A. di Rudini 8, 20142 Milan, Italy) and prof. Maurizio Sanguinetti (Università Cattolica del Sacro Cuore, Rome, Italy). The antifungal susceptibility testing was previously published by our team [18].

Both strains were preserved at −80 °C in 1 mL of yeast extract peptone dextrose broth (YPD broth, Biolife, Milan, Italy) supplemented with 30% sterile glycerol (Centralchem, Bratislava, Slovakia), from which 100 µL was added to 40 mL of YPD broth with 2% D-glucose and cultivated at 37 °C overnight on a thermal shaker at 150 RPM (Multitrone Standard, Infors HT, Basel, Switzerland). The inoculum was plated on YPD plates containing 2% D-glucose and cultivated at 37 °C overnight to obtain single colonies. The next day, one colony was taken and resuspended in 40 mL of YPD broth with 2% D-glucose and cultivated at 37 °C overnight with shaking at 150 RPM. The inoculum was later transferred to a 50 mL Falcon tube (Sarstedt AG & Co. KG, Nümbrecht, Germany) and centrifugated at 3000× *g*, 5 min, 15 °C (Universal 32R, Andreas Hettich GmbH & Co. KG, Tuttlingen, Germany). The supernatant was discarded and the pellets were washed with 40 mL of phosphate saline buffer (PBS, 137 mM NaCl, 2.7 mM KCl, 10 mM Na_2_HPO_4_, 2 mM KH_2_PO_4_, pH 7.4, all chemicals from Centralchem, Bratislava, Slovakia) and centrifugated at 3000× *g*, 5 min, 15 °C (Universal 32R, Andreas Hettich GmbH & Co. KG, Tuttlingen, Germany). The washing step was repeated two more times under the same conditions. The supernatant was discarded and the pellet was resuspended in 20 mL of PBS. The density of cells was calculated using a hemocytometer (Paul Marienfeld GmbH & Co. KG, Lauda-Königshofen, Germany) and set to 1.10^6^ cells/mL in RPMI-1640 without phenol red; it was supplemented with 2% D-glucose and buffered to pH 7.0 with 0.165 M MOPS. Biofilms were set up in a 24- or 96-well flat-bottom plate (TC Plate, Sarstedt AG & Co. KG, Nümbrecht, Germany) using 100 µL of 1 × 10^6^ cells/mL or 1 mL of 1 × 10^5^ cells/mL suspension, respectively. The plates were cultivated for 24 h at 37 °C in a thermal incubator (Thermostatic Cabinet, Lovibond, Tintometer GmbH, Dortmund, Germany).

### 2.2. MB Efficiency and PDI Assay

The stock solution of dye MB (3,7-bis(dimethylamino)-phenothiazin-5-ium chloride; Loba Feinchemie GmbH, Fischamend, Austria) was prepared by diluting 0.083 g of MB powder in 40 mL of sterile distilled water. The concentration of this solution was calculated by measuring absorbance at OD_664_ and employing the formula for molar absorption coefficient (ε = 90,000 L.mol^−1^.cm^−1^). MB solution was then filtered through a syringe filter with a pore size >0.22 μm (Syringe-filter 0.22 µm, TPP, Trasadingen, Switzerland) into a new sterile 50 mL Falcon tube and stored at 4 °C for up to 2 weeks. The tested concentrations of MB were as follows: 0.25, 0.5, 1, and 2 mM.

The effectiveness of MB was tested on a 24 h biofilm in the dark (MB Dark) to minimize interference by the PDI effect. Medium from wells with a 24 h biofilm was discarded, then 100 µL of MB of the appropriate concentration was added and incubated for 15 min, and 100 µL of PBS was added to the control sample. Then, MB was discarded by pipetting, and the biofilm was carefully washed with 100 µL of 1× PBS. The biofilm layer was scraped thoroughly with a plastic pipette tip and serially diluted. Dilutions were streaked on a YPD plate with 2% D-glucose and cultivated for 48 h at 37 °C. Owing to the small colony size, a longer cultivation period was needed to properly count colony-forming units (CFU). The results were evaluated as the percentage of viable cells treated with MB and those treated with MB, but kept in the dark, compared with the control sample (100%) without MB. Based on this experiment, the optimal concentration of MB was selected to achieve good effectiveness, but with enough surviving yeast for molecular analysis.

The preparation of 24 h biofilms and pre-cultivation with MB was carried out as described above. For this experiment, the concentration of 0.25 mM MB was selected. A previously described [19] employment of red laser light (λ = 660 nm, 190 mW/cm^2^) was used. The control group was washed with PBS alone; the other control group was treated with MB, but kept in the dark without irradiation, and the tested groups with MB were irradiated for 79 s, 120 s, and 300 s, corresponding to an energy delivery of 15, 23, and 58 J/cm^2^, respectively. As described previously, the medium from the 24 h biofilm was discarded and 100 µL of 0.25 mM MB was added to the treated groups. Then, 100 µL of PBS was added to the control group without MB, followed by a 15 min incubation. Then, MB was carefully discarded and the biofilm was irradiated with a red laser at a distance of 5 cm from the bottom of the well for the corresponding periods of irradiation. Laser focussing was adapted to the type of plate. Samples were processed as described above. The results were evaluated as the percentage of viable cells treated with MB with/without (MB Dark) irradiation compared with the control sample (100%) without MB. Biofilms were prepared in three parallel wells for each set of conditions and the experiment was repeated in at least four independent replicas. Both experiments were performed in at least four independent replicas. The data summarized in the figures represent the average value with standard deviations (±SD).

### 2.3. CLSM

Confocal laser scanning microscopy (CLSM, Zeiss LSM 510 Meta, Jena, Germany) was used to observe the PDI effect. The experiment was conducted using the same protocol described by Černáková et al. (2017) [19]. Samples were prepared in the same way as those for the 96-well plates, but 8-well chambers for microscopy (Ibidi, Martinsried, Germany) with a final volume of 200 μL were used. The samples were stained immediately after irradiation with calcofluor white (CW, Fluka, Buchs, Switzerland, excitation 355 nm/emission 433 nm) and propidium iodide (PI, Sigma Aldrich, Steinheim, Germany, excitation 535 nm/emission 617 nm). The stock solution of CW was 1 μg/mL, that of PI was 0.1 μg/mL (*w*/*v*) in deionized water, and the CW was diluted 10-fold before being added. Then, 30 μL of the dyes was administrated to the samples approximately 1 min before microscopic examination.

### 2.4. Isolation of RNA and Reverse Transcription to cDNA

RNA isolation was conducted immediately after PDI treatment. All steps prior to RNA isolation are described in paragraph 2.2. To obtain proper RNA yields, we conducted these experiments in 24-well flat-bottom plates to maximize the culmination of biofilm biomass. Before irradiation, 500 µL of 0.25 mM MB solution was added to all treated groups with/without irradiation. All remaining conditions of the PDI assay were kept the same. The irradiation period for 120 s was chosen because of a significant reduction in the survival of biofilm cells, while RNA yields were enough high. After the irradiation step, 500 µL of 1× PBS was added per well, then biofilms were scraped thoroughly with a plastic tip and transferred to a 2 mL microcentrifuge tube and centrifugated at 10,000× *g*, 2 min, 15 °C (High-Speed Microliter Centrifuge Frontier™ 5515R, Ohaus Europe GmbH, Greifensee, Switzerland), in order to wash excess MB from the pellet. After discarding the supernatant, 1 mL of PBS was added to resuspend the cells before centrifugation at 8000× *g*, 2 min, 15 °C. This washing step was performed twice, up to a maximum of three times, to obtain the clearest possible supernatant liquid. After the last wash, the supernatant was discarded and the isolation of RNA proceeded with a GeneJET RNA Purification Kit (Thermo Scientific, Waltham, MA, USA) with the following modifications: 200 µL of yeast lysis was added to resuspend the pellet followed by a 60 min incubation at 30 °C. All other steps were conducted by following the kit protocol. Eluted RNA was then purified with DNase I (Thermo Scientific, Waltham, MA, USA) and samples were stored at −80 °C or used immediately in a downstream application. To obtain cDNA for qPCR experiments, a cDNA synthesis kit was used (Maxima First Strand cDNA Synthesis Kit, Thermo Scientific, Waltham, MA, USA). Synthesized cDNA was stored at −20 °C or used immediately in qPCR.

### 2.5. Relative Change in Gene Expression Using RT-qPCR

All used primers were synthesized according to previously published sequences [20] and the *ACT1* housekeeping gene was used as the control. For initial confirmation by PCR, a thermal protocol was set up (initial denaturation at 95 °C for 10:00 min followed by 40 cycles of denaturation at 90 °C for 0:15 min, annealing at 58 °C for 1:00 min and extension at 72 °C for 1:15 min, followed by a final extension at 72 °C for 10:00 min). Gel electrophoresis was performed to confirm product lengths (2% agarose gel, 120 V, 90 min). Afterwards, we set up a thermal protocol for two-step qPCR (40 cycles of denaturation at 95 °C for 0:15 min and annealing at 58 °C for 1:00 min). For the reaction, GoTaq^®^ qPCR Master Mix (Promega Corp., Madison WI, USA) and an Mx3000P qPCR system (Agilent Technologies, Inc., Santa Clara, CA, USA) were used. All data were analysed by MxPro software provided by Agilent Technologies. Relative gene expression change was calculated using the 2^ΔΔCq^ method [21]. The *C. auris* H261 24 h biofilm was set up as an untreated control sample and normalized to a value of 1. All values are represented as log_2_ of initial values. All values are relative to the untreated control sample. The experiment was performed in three parallel wells in four independent replicas.

### 2.6. Statistical Analysis

The statistical comparisons were performed using two-way ANOVA in GraphPad Prism software (Graph Pad, San Diego, CA, USA). Dunnett’s multiple comparisons test was used to determine significant differences between groups. Differences were considered to be significant at different *p*-values: *p* < 0.05 (∗), *p* < 0.01 (∗∗), *p* < 0.001 (∗∗∗), and *p* < 0.0001 (∗∗∗∗).

## 3. Results

### 3.1. Efficiency of MB and PDI Assay

**Figure 1:**
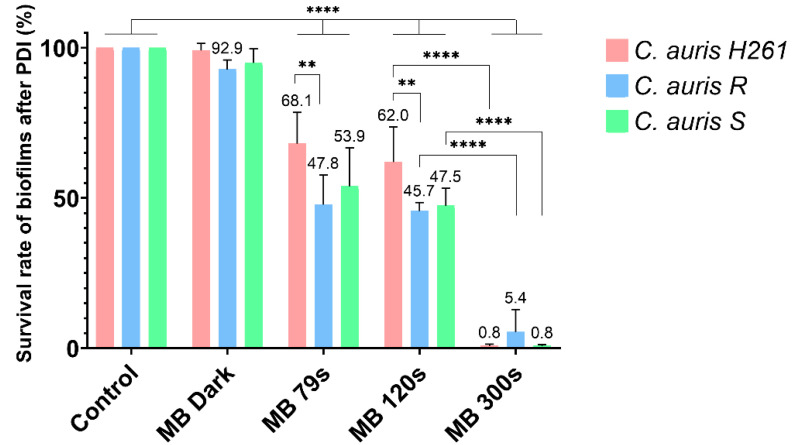
Killing effect of PDI on *C. auris* H261, *C. auris* R, and *C. auris* S. The control group represents a 24 h biofilm without MB; the MB Dark group was incubated with 1 mM MB for 1 h. The MB 79 s, MB 120 s, and MB 300 s groups were irradiated for different periods after the initial 1 h pre-incubation. The results were considered to be significant at different *p*-values: *p* < 0.05 (∗), *p* < 0.01 (∗∗), *p* < 0.001 (∗∗∗) and *p* < 0.0001 (∗∗∗∗).

Previous experiments with *C. albicans* SC5314 were performed with 1 mM MB [19]. Therefore, the experiment with *C. auris* was performed under the same conditions. However, the achieved killing effect was very high, corresponding to a 1000 times reduction in the survival of biofilm cells after a prolonged period of irradiation (300 s) compared with the control without MB. Moreover, this concentration of MB also demonstrated an antimicrobial effect in the dark (Figure 1). Similarly, 2 and 0.5 mM MB significantly reduced survival of biofilm cells, even in the dark (Supplementary data, Figure S1).

**Figure 2:**
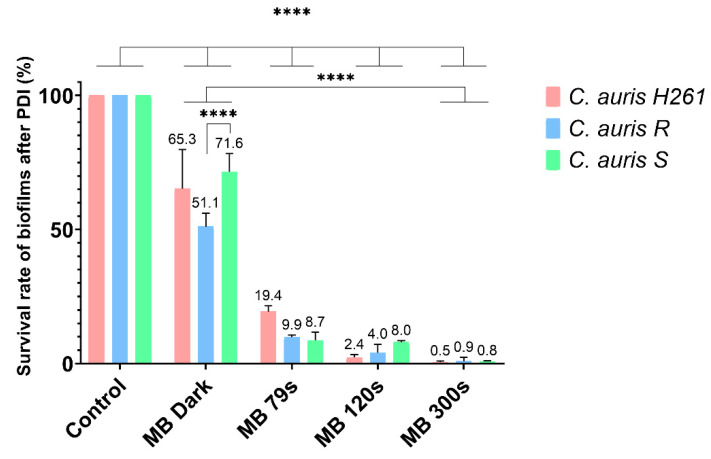
Effectiveness of PDI on *C. auris* H261, *C. auris* R, and *C. auris* S. The control group represents a 24 h biofilm without MB; the MB Dark group was incubated with 0.25 mM MB for 15 min. The MB 79 s, MB 120 s, and MB 300 s groups were irradiated for different periods after the initial 15 min pre-incubation. The results were considered to be significant at different *p*-values: *p* < 0.05 (∗), *p* < 0.01 (∗∗), *p* < 0.001 (∗∗∗) and *p* < 0.0001 (∗∗∗∗).

Another test was conducted on a 24 h biofilm in the presence of 0.25 mM MB and 15 min pre-incubation in the dark. In the MB Dark group, a minor inhibitory effect of MB was observed in all *C. auris* isolates (Figure 2). In all irradiated groups, the reduction in living cells was from 31.9% to 99.2%. A direct correlation between cell killing and the duration of irradiation was observed. No major difference was seen between the groups irradiated for 79 s and 120 s (MB 79 s and MB 120 s, respectively), while cell survival in the group irradiated for 300 s (MB 300 s) was significantly decreased (*p* ≤ 0.0001), corresponding to a 100 times reduction in cell survival compared with the control sample. This concentration of MB and 15 min pre-incubation and irradiation for 120 s were selected for experiments aimed at the expression of the efflux genes.

### 3.2. CLSM Confirmation of PDI Effect

**Figure 3:**
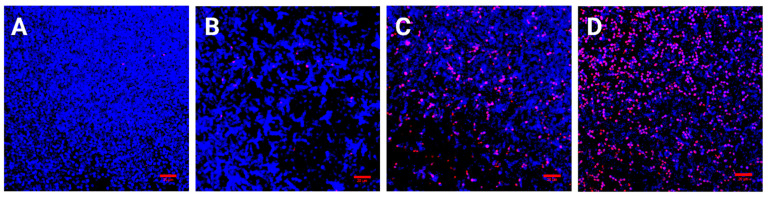
CLSM was performed on *C. auris* H261 before and after PDI in the presence of 0.25 mM MB; (**A**) control 24 h biofilm; (**B**) MB Dark group; and (**C**) MB groups irradiated for 120 s (**D**) and 300 s. Bars represent 20 µm. The cell wall was stained with CW (1 μg/mL) and dead cells were stained with PI (0.1 μg/mL).

To confirm and visualize the PDI effect on biofilm, CLSM was performed after irradiation in all strains. Figure 3 is a representative figure documenting the effect of PDI on *C. auris* H261. The cell wall of all cells was stained with CW and dead cells were stained with PI. Only a small number of dead cells were present in the MB Dark group (Figure 3B) compared to the control group (Figure 3A). However, *C. auris* irradiated for 120 s exhibited a higher number of dead cells (Figure 3C), while irradiation for 300 s confirmed a prominent killing effect (Figure 3D). Similar results were observed with both resistant strains of *C. auris* (Supplementary data, Figure S2).

### 3.3. Relative Change in Gene Expression of Efflux Transporters

**Figure 4:**
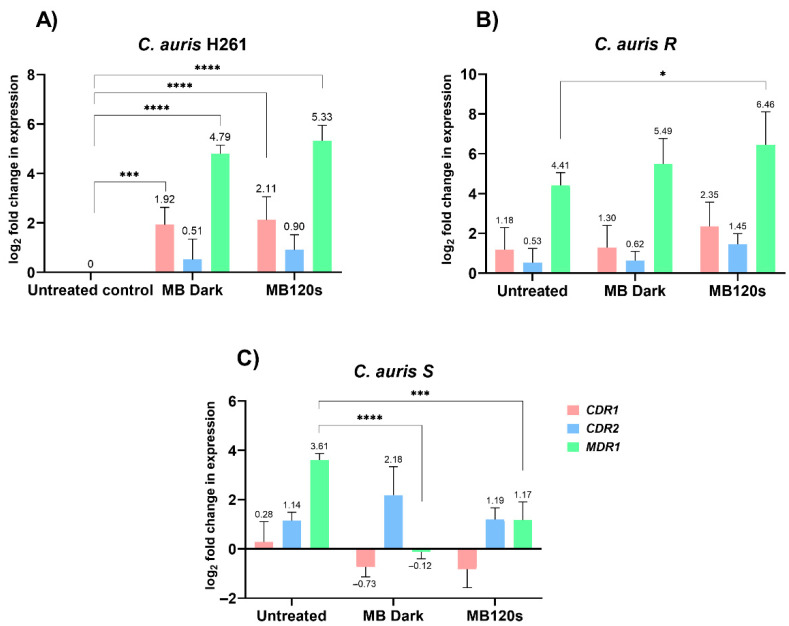
Log_2_ fold change in expression of *CDR1*, *CDR2*, and *MDR1* genes in 24 h biofilms of sensitive strain *C. auris* H261 (**A**) and resistant strains of *C. auris* R (**B**) and *C. auris* S (**C**). The expression of genes was normalized to the housekeeping gene *ACT1*. The untreated control was a 24 h biofilm without MB of *C. auris* H261, set to a value of 1 for all tested genes, and all respective values were calculated relative to this value. The untreated groups were a 24 h biofilm without MB, the MB Dark group was incubated with 0.25 mM MB, and MB 120 s was the group treated with 0.25 mM MB and irradiated for 120 s. The results were considered to be significant at different *p*-values: *p* < 0.05 (∗), *p* < 0.01 (∗∗), *p* < 0.001 (∗∗∗) and *p* < 0.0001 (∗∗∗∗).

FLU-resistant strains of *C. auris* R and *C. auris* S (Figure 4) manifested upregulation of the *CDR1* and *MDR1* genes in 24 h biofilms compared to the untreated control sample represented by a biofilm of sensitive strain *C. auris* H26, set to a value of 1. The presence of 0.25 mM MB affected the expression of the *MDR1* gene in all tested samples. The most prominent change was observed in resistant *C. auris* R (Figure 4B) after irradiation, with more 6.46-fold changes in expression. Up-regulation of 5.33-fold was determined by *C. auris* H261 (Figure 4A). Upregulation of the *MDR1* gene was also observed in the MB Dark groups with an over 4.71-fold and 5.49-fold increase for *C. auris* H261 and *C. auris R*, respectively. Moreover, changes in the *MDR1* gene expression were observed in *C. auris* S (Figure 4C) with decreased expression in the MB Dark group, with a lower effect in the MB 120 s group. The *CDR1* gene also exhibited upregulation in both the irradiated and MB Dark groups, mostly in *C. auris* H261 and *C. auris* R; however, downregulation was observed in the MB Dark and MB 120 s groups in *C. auris* S. Expression of the *CDR2* gene did not show any relevant changes in any of the groups.

## 4. Discussion

*C. auris* is an emerging fungal pathogen of the last decade that exhibits resistance to multiple drugs, including the most prescribed antifungal, FLU [2]. PDI manifests the ability to inhibit growth [19,22], completely eradicate fungal biofilms, and boost antifungal efficacy [23], which have been well documented *in vitro*, *in vivo*, and *ex vivo* [24,25,26]. With *C. auris*, more data are needed to properly assess the effectiveness of PDI, as only a limited number of studies have been published [27,28]. Both studies showed great potential to inhibit *C. auris* biofilm in the presence of MB. Undoubtedly, PDI represents a novelty in the therapy of fungal disease with many advantages, mainly owing to resistance in microbes [14]. Similarly to photodynamic therapy, the limitation of PDI is mainly due to lower selectivity, the unfavourable biodistribution of PS, and long-lasting skin sensitivity to light [29,30].

In this study, three *C. auris* isolates were tested. Previously published work by our group confirmed that *C. auris* S and *C. auris* R manifested a markedly decreased susceptibility/resistance to FLU, whereas *C. auris* H261 was susceptible to FLU as well as to both antifungal drugs of caspofungin and amphotericin B [18].

MB-based PDI exhibited significant inhibition in terms of the survival of biofilms formed by both sensitive and resistant strains of *C. auris*, while no major difference in susceptibility to PDI was observed in 24 h biofilms formed by both resistant isolates compared with the sensitive strain. The obtained data proved that the inhibitory effect can be increased by either an increase in MB concentration and/or a prolonged period of irradiation. The effect can be increased to a more than 99.9% inhibitory effect compared to the control sample, corresponding to an almost !1000 times reduction in the survival of biofilm cells. However, for the determination of changes in gene expression, experimental conditions were chosen that produced a significant reduction in the survival of biofilm cells while maintaining sufficient RNA robustness.

CLSM results supported the observations on PDI outcome, with a distinguishable difference between the treated and non-treated groups. Visible, red-stained nuclei of dead cells suggested that ROS could effectively kill cells in a time-dependent manner.

A similar study to ours was conducted by Tan et al. (2019) [28], in which the *in vitro* effect of PDI using MB combined with LED light on the viability of planktonic cells and biofilms of five clinical strains of *C. auris* was observed. MB of different concentrations (8, 16, and 32 μg/mL) was applied as the PS and an LED (λ = 635 nm, 12 and 24 J/cm^2^) device was used as a light source. The results showed that there was no growth of the tested *C. auris* strains after PDI planktonic cultures. In addition, PDI exhibited a colony-forming unit reduction of up to 7.20 log_10_ against *C. auris* biofilms. These data demonstrate that *in vitro* PDI using MB irradiated with a red LED source offers promising potential for the treatment of *C. auris* infections [28]. This paper’s results also support this evidence on the inhibition of biofilm cells.

The main hypothesis of this manuscript is based on the questions of (i) whether and in what way PDI is related to the activity of efflux pumps and (ii) whether the effectiveness of PDI could work despite the activity of efflux transporters.

It was hypothesized that the source of this difference in susceptibility to PDI can be attributed to the efflux effect, as previous work demonstrated that MB is a substrate for Mdr1 [17]. MB can be taken up by cells through assisted diffusion, but the efflux transporters probably play a major role in its excretion by cells, which may lead to a lower susceptibility to PDI. The concentration of MB used also plays a role.

The efflux pump activity of *C. auris* was assessed by Kean et al. (2018) [10] using the transcriptome assay and the measurement of fluorescence by alanine-naphthylamine fluorescent assay. The results proved an increased activity of efflux pumps associated with resistance within *C. auris* communities in a 12 and 24 h biofilm compared with planktonic cells. When these were inhibited, FLU sensitivity was enhanced 4- to 16-fold. This study demonstrated and proposed the significance of the efflux-mediated resistance of *C. auris* to a range of antimicrobial agents within hospital settings. In our previous study, we confirmed a high native upregulation of the efflux genes, specifically *CDR1*, but mainly *MDR1* in both resistant *C. auris* strains [18]. After PDI, upregulation of the *MDR1* and *CDR1* genes was observed in the susceptible strain *C. auris* H261 and in FLU-resistant *C. auris R*. However, decreased regulation was observed in the second resistant strain, *C. auris* S. Regulation of the *CDR2* gene was not relevant to PDI. The presented findings do not agree with the observations of Spring et al. (2015) [31] and described by Chu et al. (2020) [13], in which a surprising effect of PDI in cancer cells was observed; PDI-induced ROS production directly damaged selected proteins, concretely some of the ABC transporters, which can diminish multidrug resistance [13,31]. Taking into account our findings, we can assume that the overregulation of the efflux genes during PDI does not necessarily mean that the population of *Candida* cells becomes resistant.

## 5. Conclusions

In this work, the observed upregulation of the *MDR1* and *CDR1* genes did not affect the overall efficacy of MB-mediated PDI on biofilms formed by *C. auris* clinical isolates, regardless of their sensitivity or resistance to antifungals. These findings should be investigated further to assess a possible increase in resistance to antifungals in the surviving biofilm’s sub-population through the upregulation of efflux genes, namely *MDR1* and *CDR1*. As the efficacy of PDI can be increased by higher concentrations of MB or prolonged irradiation, it is strongly predicted that PDI’s effect can overcome the efflux of *Candida* spp.

## Data Availability

Not applicable.

## Supplementary Materials

The following supporting information can be downloaded at www.mdpi.com/article/10.3390/jof8111137/s1. Figure S1: Additional testing of MB concentrations on *C. auris* H261, *C. auris* R and *C. auris* S in dark conditions; Figure S2: CLSM performed on S and R before and after PDI in the presence of 0.25 mM MB.
