# Supplementary material for: Photodynamic Inactivation Effectively Eradicates Candida auris Biofilm despite Its Interference with the Upregulation of CDR1 and MDR1 Efflux Genes
Source: J Fungi (Basel). 2022 Oct 27;8(11):1137. doi: 10.3390/jof8111137 (PMC9697027; doi:10.3390/jof8111137)
Supplement: Supplementary file 1 [file jof-08-01137-s001.zip › jof-1960389-supplementary.pdf]

Photodynamic inactivation effectively eradicates *Candida auris* biofilm despite its interference with the upregulation of *CDR1* and *MDR1* efflux genes

Matúš Štefánek[1], Lucia Černáková[1], Jaroslava Dekkerová[1] and Helena Bujdáková[1,*]

[1] Department of Microbiology and Virology, Faculty of Natural Sciences, Comenius University in Bratislava, 842 15 Bratislava, Slovakia

* Correspondence: helena.bujdakova@uniba.sk; Tel.: +421-2-9014-9436, https://orcid.org/0000-0002-9219-0803

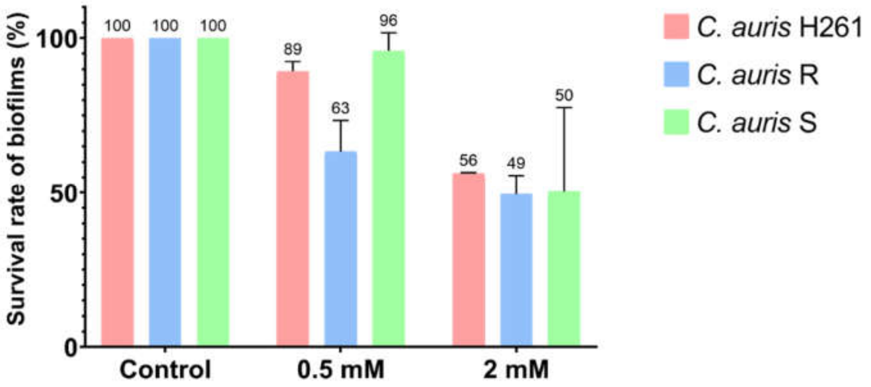

Figure S1. Effectiveness of 2 and 0.5 mM MB in dark on *C. auris* H261, *C. auris* R and *C. auris* S.

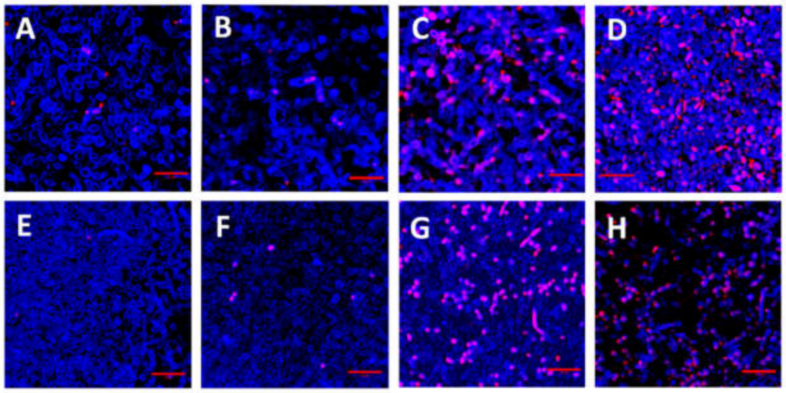

Figure S2. CLSM was performed on *C. auris* S (top row, **A-D**) and *C. auris* R (bottom row, **E-H**) before and after PDI in the presence of 0.25 mM MB; control 24-h biofilms (**A, E**); MB Dark groups (**B, F**); MB groups irradiated for 120s (**C, G**); and 300s (**D, H**). Bars represent 20 μm. The cell wall was stained with CW (1 μg/mL) and dead cells were stained with PI (0.1 μg/mL).
